# Harmful and Beneficial Role of ROS 2017

**DOI:** 10.1155/2018/5943635

**Published:** 2018-04-02

**Authors:** Sergio Di Meo, Tanea T. Reed, Paola Venditti, Victor M. Victor

**Affiliations:** ^1^Dipartimento di Biologia, Università di Napoli “Federico II”, 80126 Napoli, Italy; ^2^Department of Chemistry, Eastern Kentucky University, Richmond, KY 40475, USA; ^3^Service of Endocrinology, Dr. Peset University Hospital, Foundation for the Promotion of Health and Biomedical Research in the Valencian Region (FISABIO), 46010 Valencia, Spain; ^4^Department of Physiology, University of Valencia, Valencia, Spain

Over the years, the idea of a contrasting dual role played by free radicals in living organisms has become increasingly validated. On the one hand, a look at the recent literature confirms that free radicals are oxidizing agents implicated in tissue damage and that uncontrolled oxidative activity underlies various pathological conditions and can be countered by antioxidant treatment. On the other hand, research shows that oxidants exert beneficial effects by regulating cell signaling cascades. Further support for the idea of harmful and beneficial roles of free radicals is provided by the articles in the present special issue.

One paper examines extracellular thioredoxins, small oxidoreductase enzymes that control redox homeostasis in the cell via NADPH, which is generated by the pentose phosphate pathway as a cofactor of thioredoxin reductase. T. Léveillard and N. Aït-Ali chronologically reviewed key findings concerning extracellular thioredoxins. Starting with the discovery of human thioredoxin (TXN1), first identified as the adult T-cell leukemia-derived factor (ADF), a secreted protein, they go on to examine the extracellular truncated thioredoxin RdCVF encoded by the *NXNL1* gene, a unique example of a complete extracellular thioredoxin signaling system.

Several papers deal with the damaging effects of ROS and, in some cases, the protective effects of antioxidant intervention. The work by M. Cebova et al. shows that the polyphenol-rich extract of *Aronia melanocarpa* has a positive effect on blood pressure, NO-synthase activity, and proinflammatory processes in experimentally induced hypertension, a condition normally associated with endothelial dysfunction and oxidative stress.

I. V. Chestkov et al. show that the leukocytes of male paranoid schizophrenia patients contain more mitochondrial DNA (mtDNA) than those of both male patients treated with antipsychotic medication and healthy controls. Furthermore, the mtDNA content of unmedicated patients positively correlates with the level of 8-oxodG, a marker of DNA oxidation.

M. Herbet et al. studied biochemical and molecular changes associated with ROS generation in the brains of rats submitted to variable levels of chronic environmental stress. Their results show that this stress causes lipid and DNA oxidative damage and disruption of the antioxidant defense system, including decreased expression of gene encoding antioxidative transcriptional factors. Furthermore, they detected activation of oxidative stress-responsive genes such as 8-oxoguanine glycosylase1 and methionine sulfoxide reductase A, which play a role in triggering the oxidative DNA repair system.

L. Subedi et al. evaluated the efficacy of the antiaging compound resveratrol contained in genetically modified normal edible rice as a treatment for skin aging caused by chronic exposure to ultraviolet radiation. They found that this resveratrol-enriched rice overcomes the usual drawbacks of resveratrol and enhances its antiaging potential by controlling major pathways of skin aging.

H. Liu et al. investigated whether decreased AChE activity during sepsis is related to oxidative stress by observing AChE activity in different grades of sepsis induced by caecal ligation and puncture. Their results show that AChE activity at the neuromuscular junction of the diaphragm decreases more significantly during severe sepsis. Furthermore, this activity is significantly and negatively correlated with the level of oxidative stress during sepsis.

The review by B. P. Mihalas et al. explores the reduced capacity of the aging oocyte to mitigate macromolecular damage arising from oxidative insults and highlights the dramatic consequences for oocyte quality and female fertility. It discusses how impaired ROS metabolism, decreased DNA repair, reduced sensitivity of the spindle assembly checkpoint, and decreased capacity for protein repair and degradation collectively render the aged oocyte acutely vulnerable to oxidative stress.

G. Pizzino et al. studied glycemic control and oxidative stress markers in male adolescents with increased urinary levels of cadmium. Their results indicate that cadmium burden alters glycemic control in adolescents and that oxidative stress plays a key role in cadmium-induced insulin resistance, thus increasing the risk of developing metabolic disorders.

G. Rowicka et al. evaluated the presence of oxidative stress in obese prepubertal children. They found a significant negative correlation between total antioxidant capacity and ox-LDL concentrations. Furthermore, obesity duration was positively correlated with total oxidative capacity level, which suggests that obesity-related oxidative stress already occurs in prepubescence.

The review by G. Scutiero et al. focuses on how a disruption of the balance between ROS production and antioxidant defenses and the oxidative stress, occuring as a consequence, affects the development and progression of endometriosis. The study of aspects such as iron metabolism, oxidative stress markers, genes involved in oxidative stress, endometriosis-associated infertility, and cancer development supports the role of oxidative stress in the development and progression of endometriosis.

A. N. Onyango reviews the literature which suggests that insulin-responsive cells such as endothelial cells, hepatocytes, adipocytes, and myocytes also produce singlet oxygen, thus contributing to insulin resistance, for example, by generating bioactive aldehydes, inducing endoplasmic reticulum (ER) stress, and modifying mitochondrial DNA.

One paper by N. Cichoń et al. examines the beneficial effect of nitrogen radicals such as nitric oxide (NO^•^). Indeed, it investigated the effect of an extremely low-frequency electromagnetic field (ELF-EMF) on the generation and metabolism of NO^•^ as a neurotransmitter on the rehabilitation of poststroke patients. They observed that the application of ELF-EMF significantly increased 3-nitrotyrosine and nitrate/nitrite levels, while *NOS2* expression was insignificantly decreased. Their results showed that ELF-EMF treatments also improve functional and mental status. The authors conclude that ELF-EMF therapy can promote recovery in poststroke patients.

Two other reviews deal with harmful and beneficial roles of ROS. A. L. Santos et al. summarize recent advances in research in the field and show that this dual role is found across species, from bacteria to humans, and in various aspects of cellular physiology. They also highlight the utility of bacterial models to elucidate the molecular pathways by which ROS mediate aging and aging-related diseases. G. Pizzino et al. describe recent findings regarding oxidative stress, highlighting both its positive and negative influence on human health. The authors show that oxidative stress and free radicals are generally detrimental to human health, contributing to the initiation and progression of several pathologies ranging from cardiovascular disease to cancer. On the other hand, they discuss how some prooxidant compounds or agents can benefit human health, particularly regarding cancer treatment.

*Sergio Di Meo* *Tanea T. Reed* *Paola Venditti* *Victor M. Victor*

